# Modeling and Experimental Demonstration of a Hopfield Network Analog-to-Digital Converter with Hybrid CMOS/Memristor Circuits

**DOI:** 10.3389/fnins.2015.00488

**Published:** 2015-12-24

**Authors:** Xinjie Guo, Farnood Merrikh-Bayat, Ligang Gao, Brian D. Hoskins, Fabien Alibart, Bernabe Linares-Barranco, Luke Theogarajan, Christof Teuscher, Dmitri B. Strukov

**Affiliations:** ^1^Department of Electrical and Computer Engineering, University of California, Santa BarbaraSanta Barbara, CA, USA; ^2^Centre National de la Recherche ScientifiqueLille, France; ^3^Instituto de Microelectronica de Sevilla (Consejo Superior de Investigaciones Científicas and University of Seville)Seville, Spain; ^4^Department of Electrical and Computer Engineering, Portland State UniversityPortland, OR, USA

**Keywords:** Hopfield network, recurrent neural network, hybrid circuits, memristor, resistive switching, analog-to-digital conversion

## Abstract

The purpose of this work was to demonstrate the feasibility of building recurrent artificial neural networks with hybrid complementary metal oxide semiconductor (CMOS)/memristor circuits. To do so, we modeled a Hopfield network implementing an analog-to-digital converter (ADC) with up to 8 bits of precision. Major shortcomings affecting the ADC's precision, such as the non-ideal behavior of CMOS circuitry and the specific limitations of memristors, were investigated and an effective solution was proposed, capitalizing on the in-field programmability of memristors. The theoretical work was validated experimentally by demonstrating the successful operation of a 4-bit ADC circuit implemented with discrete Pt/TiO_2−__*x*_/Pt memristors and CMOS integrated circuit components.

## Introduction

Recurrent artificial neural networks are an important computational paradigm capable of solving a number of optimization problems (Hopfield, 1984; Tank and Hopfield, 1986). One classic example of such networks is a Hopfield analog-to-digital converter (Tank and Hopfield, 1986; Lee and Sheu, 1989; Smith and Portmann, 1989). Although such a circuit may be of little practical use, and inferior, for example, to similar-style feed forward-type ADC implementations (Chigusa and Tanaka, 1990), it belongs to a broader constrained optimization class of networks which minimize certain pre-programmed energy functions and have several applications in control and signal processing (Tank and Hopfield, 1986). The Hopfield network ADC circuit also represents an important bridge between computational neuroscience and circuit design, and an understanding of the potential shortcomings of such a relatively simple circuit is therefore important for implementing more complex recurrent neural networks.

**Figure 1:**
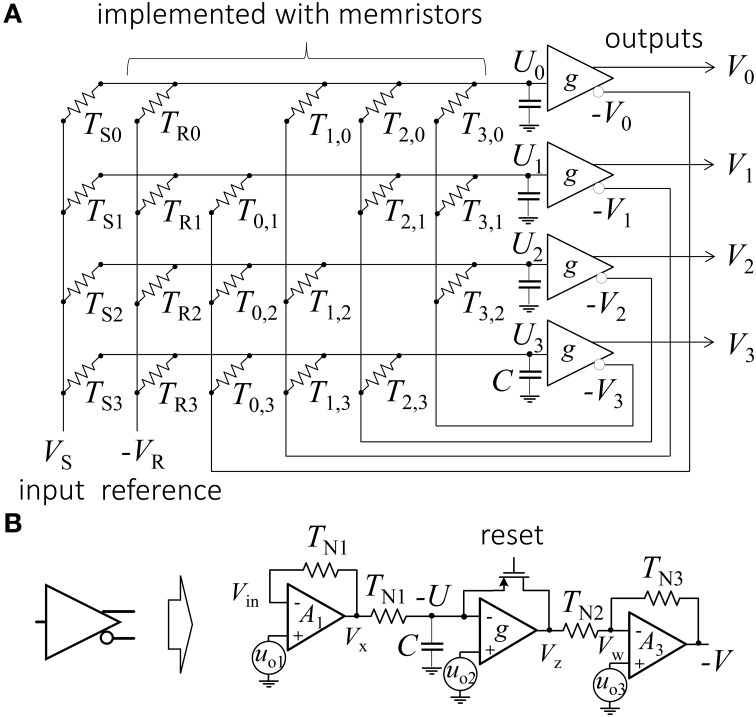
**(A)** Conventional Hopfield network implementation of a 4-bit ADC and **(B)** specific implementation of a neuron as considered in this paper.

An example of a 4-bit Hopfield network ADC is shown in Figure 1 (Tank and Hopfield, 1986). The originally proposed network consists of an array of linear resistors (also called *weights* or *synapses*) and four peripheral inverting amplifiers (*neurons*). Each neuron receives currents from the input and reference lines and from all other neurons via corresponding synapses. The analog input voltage *V*_S_ is converted to the digital code *V*_3_*V*_2_*V*_1_*V*_0_, i.e.,

(1) $$\begin{array}{r} V_{\text{S}}=\overset{3}{\underset{i=0}{\sum }}2^{i}V_{i} \end{array}$$

by first forcing all neuron outputs to zero (Lee and Sheu, 1989) and then letting the system evolve to the appropriate stationary state.

To understand how the Hopfield network performs the ADC operation, let us first describe its electrical behavior. Assuming leakage-free neurons with infinite input and zero output impedances, the dynamic equation governing the system evolution of the input voltage *U*_*j*_ of the *j*-th neuron is described as:

(2) $$\begin{array}{rcl} C{\dot{U}}_{j} & = & -\underset{i}{\sum }T_{ij}V_{i}-T_{j}U_{j}+I_{j} \end{array}$$

(2a) $$\begin{array}{rcl} V_{i} & = & g\left(U_{i}\right), \end{array}$$

where *g*(·) is a neuron activation function, *C* is the neuron's input capacitance, *T*_*ij*_ is a conductance of the synapse connecting the output of the *i*-th neuron with the input of the *j*-th neuron, while

(3) $$\begin{array}{rcl} I_{j} & = & T_{\text{S}j}V_{\text{S}}-T_{\text{R}j}V_{\text{R}}, \end{array}$$

(4) $$\begin{array}{rcl} T_{j} & = & T_{\text{S}j}+T_{\text{R}j}+\Sigma_{i}T_{ij} \end{array}$$

are the corresponding effective offset input current and effective input conductance for the *j*-th neuron. Here*V*_R_ is a reference voltage, while *T*_R_ and *T*_S_ are conductances of reference and input weights, respectively (Figure 1A). Note that neuron input *U*_*i*_ can be either positive or negative, but the output of the neuron is either zero or positive. The inverted outputs of the neurons, which are fed back to the network, are therefore either negative or zero. One activation function suitable for such mapping is the sigmoid function 1/(1+exp[-*U*]). Neuron output needs to be inverted to keep the feedback weights positive and thus to allow physical implementation with passive devices, such as resistors^1^.

Alternatively, the Hopfield network operation can be described by an energy function. The evolution of the dynamic system described by Equation (2) is equivalent to a minimization of the energy function:

(5) $$\begin{array}{r} E=\frac{1}{2}\sum_{ij}T_{ij}V_{i}V_{j}-\sum_{j}V_{j}I_{j}-\sum_{j}T_{j}\int_{0}^{V_{j}}g^{-1}\left(V\right)dV \end{array}$$

where the last term can be neglected for very steep transfer functions (Hopfield, 1984). In Tank and Hopfield (1986), showed that a 4-bit ADC task (Equation 1) can be described by the following energy function:

(6) $$\begin{array}{r} E=\frac{1}{2}{\left(V_{S}-\overset{3}{\underset{i=0}{\sum }}2^{i}V_{i}\right)}^{2}-\frac{1}{2}\overset{3}{\underset{i=0}{\sum }}2^{2i}V_{i}\left(V_{i}-1\right) \end{array}$$

Here the first term tends to satisfy Equation (1), while the second tends to force each digital output *V*_*i*_ to be either “0” or “1.” After rearranging the terms in Equation (6) and comparing the result with Equation(5), the appropriate weights for performing the ADC task are:

(7) $$\begin{array}{r} T_{ij}=2^{\left(i+j\right)},T_{\text{S}j}=2^{j},T_{\text{R}j}=2^{\left(2j-1\right)}. \end{array}$$

In the Hopfield ADC network, the number of synapses grows quadratically with the number of neurons. Compact implementation of the synapses is therefore required if such circuits are to be practical. This is certainly challenging to achieve with conventional CMOS technology, because, according to Equation (7), it requires analog weights with a relatively large dynamic range, i.e., in the order of 2^2^^*N*^, where *N* is the bit precision. Weights can be stored digitally, but this approach comes with a large overhead (Moopenn et al., 1990). On the other hand, analog CMOS implementations of the synapses have to cope with the mismatch issues often encountered in CMOS circuits (Indeveri et al., 2011). Consequently, several attempts have been made to implement synapses with alternative, nonconventional technologies. In some of the early implementations of Hopfield networks, weights were realized as corresponding thin film (Jackel et al., 1987) or metal line (Graf et al., 1986; Schwartz et al., 1987) conductance values, patterned using e-beam lithography and reactive-ion-etching. The main limitation of these approaches was that the weights were essentially one-time programmable, with rather crude accuracy. A much more attractive solution was very recently demonstrated in Eryilmaz et al. (2014), which describes a Hopfield network implementation with synapses based on phase change memory paired with conventional field-effect transistors. That work, together with other recent advances in device technologies (Wu et al., 2012; Zhang et al., 2012) revived interest in the theoretical modeling of recurrent neural networks based on hybrid circuits (Waser et al., 2009; Strukov and Kohlstedt, 2012; Lehtonen et al., 2014; Rakkiyappan et al., 2014; Walls and Likharev, 2014).

This paper explores the implementation of synapses with an emerging, very promising type of memory devices, namely metal-oxide resistive switching devices (“memristor”) (Wu et al., 2012; Zhang et al., 2012). In the next section we discuss the general implementation details of the Hopfield network ADC, including the memristor devices which were utilized in the experimental setup. This is followed by a theoretical analysis of the considered hybrid circuits' sensitivity to certain representative sources of non-ideal behavior and discussion of a possible solution to such problems. The theoretical results were validated with SPICE simulations (Section Simulation Results) and experimental work (Section Experimental Results). The paper concludes with a Discussion section. It should be noted that preliminary experimental results, without any theoretical analysis, were reported earlier in Gao et al. (2013a), where we first presented a Hopfield network implementation with metal-oxide memristors. The only other relevant experimental work on memristor-based Hopfield networks that we are aware of was published recently in Hu et al. (2015). However, the network demonstrated in Hu et al. (2015) was based on 9 memristors whereas the circuit presented in this work involves 16.

## Materials and methods for Hopfield network implementation with hybrid circuits

Following on from our earlier works (Alibart et al., 2013; Gao et al., 2013b; Merrikh-Bayat et al., 2014), we here consider the implementation of a hybrid CMOS/memristive circuit (Figure 1). In this circuit, density-critical synapses are implemented with Pt/TiO_2−x_/Pt memristive devices, while neurons are implemented by CMOS circuits.

In their simplest form, memristors are two-terminal passive elements, the conductance of which can be modulated reversibly by applying electrical stress. Due to the simple structure and ionic nature of their memory mechanism, metal-oxide memristors have excellent scaling prospects, often combined with fast, low energy switching and high retention (Strukov and Kohlstedt, 2012). Many metal oxide based memristors can also be switched continuously, i.e., in analog manner, by applying electrical bias (current or voltage pulses) with gradually increasing amplitude and/or duration.

**Figure 2:**
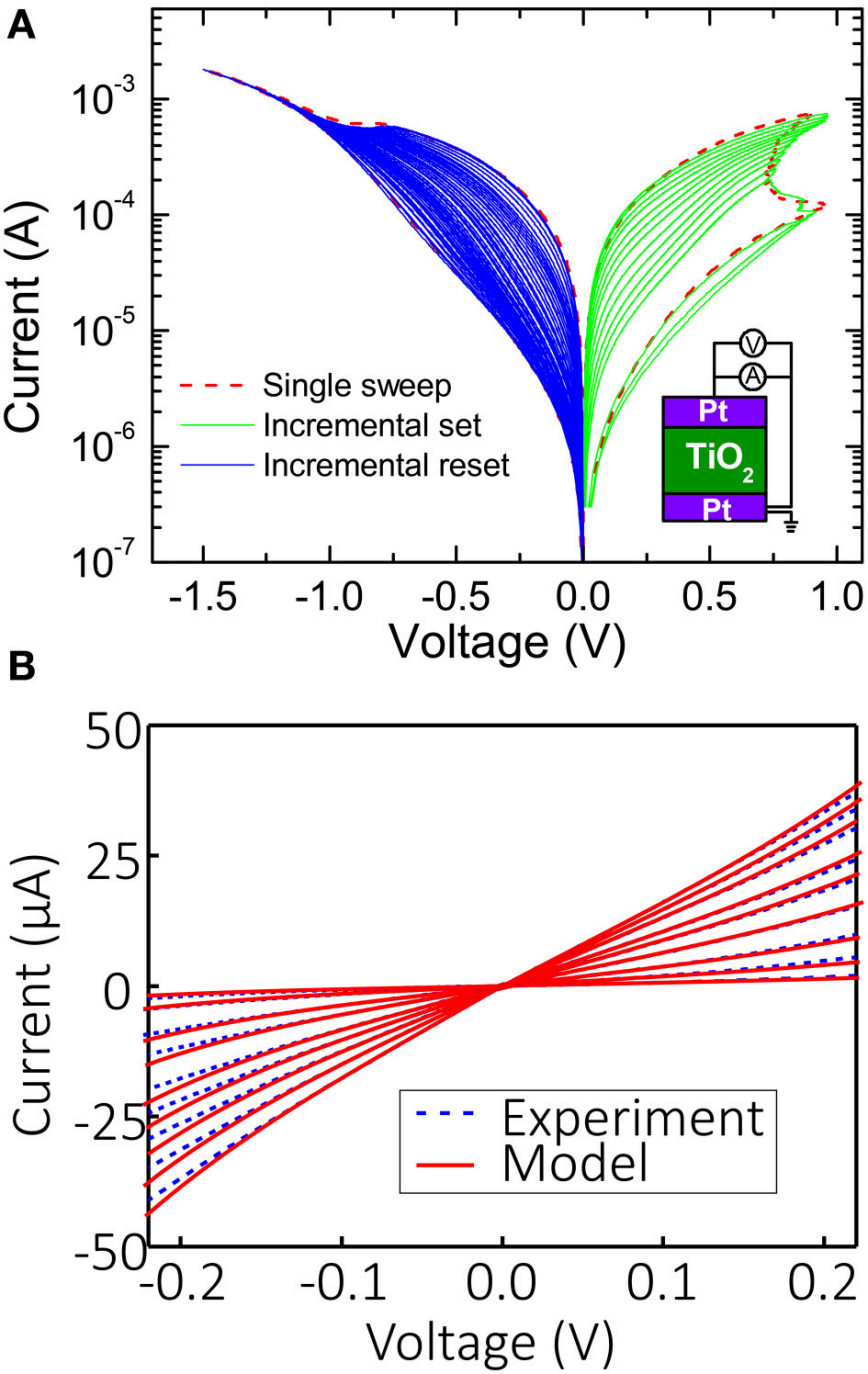
**(A)** Typical *I-V* curves with current-controlled set and voltage-controlled reset switching for the considered Pt/TiO_2−x_/Pt memristors. **(B)** Modeling of static *I-V* curves at small disturb-free voltages for several different states. The fitting parameters are β = 1, α_1_ = 14.7 V^−3^, $\alpha_{2}=-5.9\times 10^{4}$ ΩV^−3^,$\alpha_{3}=1.5\times 10^{8}$ Ω^2^V^−3^ for *V* > 0, and α_1_ = 34.6 V^−3^, $\alpha_{2}=-1.9\times 10^{5}$ ΩV^−3^, $\alpha_{3}=3.65\times 10^{8}$ Ω^2^V^−3^ for *V* < 0.

Figure 2A shows typical continuous switching *I-V*s for the considered Pt/TiO_2−x_/Pt devices (Alibart et al., 2012). The devices were implemented in “bone-structure” geometry with an active area of ~1 μm^2^ using the atomic layer deposition technique. An evaporated Ti/Pt bottom electrode (5 nm/25 nm) was patterned by conventional optical lithography on a Si/SiO_2_ substrate (500 μm/200 nm, respectively). A 30 nm TiO_2_ switching layer was then realized by atomic layer deposition at 200°C using Titanium Isopropoxide (C_12_H_28_O_4_Ti) and water as precursor and reactant, respectively. A Pt/Au electrode (15 nm/25 nm) was evaporated on top of the TiO_2_ blanket layer, and the device was finally rapidly annealed at 500°C in an N_2_ and N_2_+O_2_ atmosphere for 5 min to improve the crystallinity of the TiO_2_ material. Details of the fabrication and characterization of the considered memristors are given in Alibart et al. (2012).

After programming the memristors to the desired resistance, it was important for their state to remain unchanged during operation of the Hopfield network, so to prevent any disturbance the voltage drop across them was always kept within the |*V*| ≤ 0.2 V “disturb-free” range (Alibart et al., 2012).

The static *I-V* characteristics (i.e., those within disturb-free regime) for several different memory states are shown in Figure 2B. To assist SPICE simulation, the experimental *I-V* curves at small biases were fitted by the following static equation with a single memory state *G*:

(8) $$\begin{array}{r} I=GV+\beta \left(\alpha_{1}G+\alpha_{2}G^{2}+\alpha_{3}G^{3}\right)V^{4}. \end{array}$$

The need to keep the voltage drop across memristive devices small also affects neuron design. A simple leaky operational amplifier (op-amp) integrator could be sufficient to implement neuron functionality, but ensuring disturb-free operation with such a design is not easy. This issue was resolved by implementing neurons with three op-amps connected in series (Figure 1B). The first op-amp was an inverting amplifier which held virtual ground even if the neuron's output was saturated. The second op-amp was an open loop amplifier implementing a sign-like activation function. The field effect transistor in the negative feedback of this op-amp was initially turned on to force the neuron's outputs to zero (i.e., to set into initial state before computing output) and then turned off during network convergence. The last op-amp inverted the signal and ensured that the neuron output was within the −0.2 V ≤ *V* ≤ 0 voltage range. Note that since the neuron bandwidth was mainly determined by the input capacitance of the second amplifier, and the other sources of parasitic capacitance could be neglected for simplicity, the capacitive load of the second amplifier (Figure 1B) was effectively a neuron input capacitance (Figure 1A).

Assuming ideal op-amps and no possibility of saturation by the first and last amplifiers, the dynamic equation for this neuron design can be written as:

(9a) $$\begin{array}{rcl} C{\dot{U}}_{j} & = & -\underset{i}{\sum }T_{ij}V_{i}-T_{\text{N}1}U_{j}+I_{j} \end{array}$$

(9b) $$\begin{array}{rcl} V_{j} & = & -T_{\text{N}2}∕T_{\text{N}3}g\left(U_{j}\right), \end{array}$$

where *g*() is a transfer function of the second op-amp (see Appendix for more details on derivation).

For a very steep transfer function, the second term in the right hand part of Equation (9a) can be neglected (Hopfield, 1984). The network is then described by the original energy function (Equation 5) and the weights are proportional to those defined in Equation (7), i.e.,

(10) $$\begin{array}{r} T_{ij}’=5T_{ij},T_{\text{S}j}’=T_{\text{S}j},T_{\text{R}j}’=5T_{\text{R}j}, \end{array}$$

where the additional coefficient 5 is due to the reduced, i.e., 0.2 V, output voltage corresponding to digital “1” in the considered circuit [as opposed to output voltage 1 V assumed in the original ADC energy function in Equation (6) for ADC and the weights in Equation (7) derived from that energy function].

The physical implementation of this Hopfield network ADC posed a number of additional challenges. However, it should first be mentioned that variations in neuron delay and input capacitances, which may result in oscillatory behavior and the settling in of false energy minima (Lee and Sheu, 1989; Smith and Portmann, 1989), were not a problem in our case thanks to the slow operating speed, which was enforced to reduce capacitive coupling. The specific problems regarding the considered implementation were offsets in virtual ground, resulting from the voltage offsets (*u*_*o*_) and limited gain (*A*) of the op-amps (Figure 1B). Another, somewhat less severe, problem was the nonlinear conductance of the memristive devices (defined via parameter β–, see Equation 8). In the Appendix it is shown how limited gain and non-zero offset result in an additional constant term *I*_0_ in dynamical equation (Equation A7), which can be factored into the reference weights as follows:

(11) $$\begin{array}{r} T_{\text{R}j}”=T_{\text{R}j}’+I_{0j}∕V_{\text{R}}. \end{array}$$

The Hopfield network with practical, non-ideal neurons can still therefore be approximated by the original energy equation and it should be possible to circumvent the effects of limited gain and voltage offset by fine-tuning the reference weights. This idea was verified via SPICE modeling and experimental work, as described in the next section.

## Results

### Simulation results

**Figure 3:**
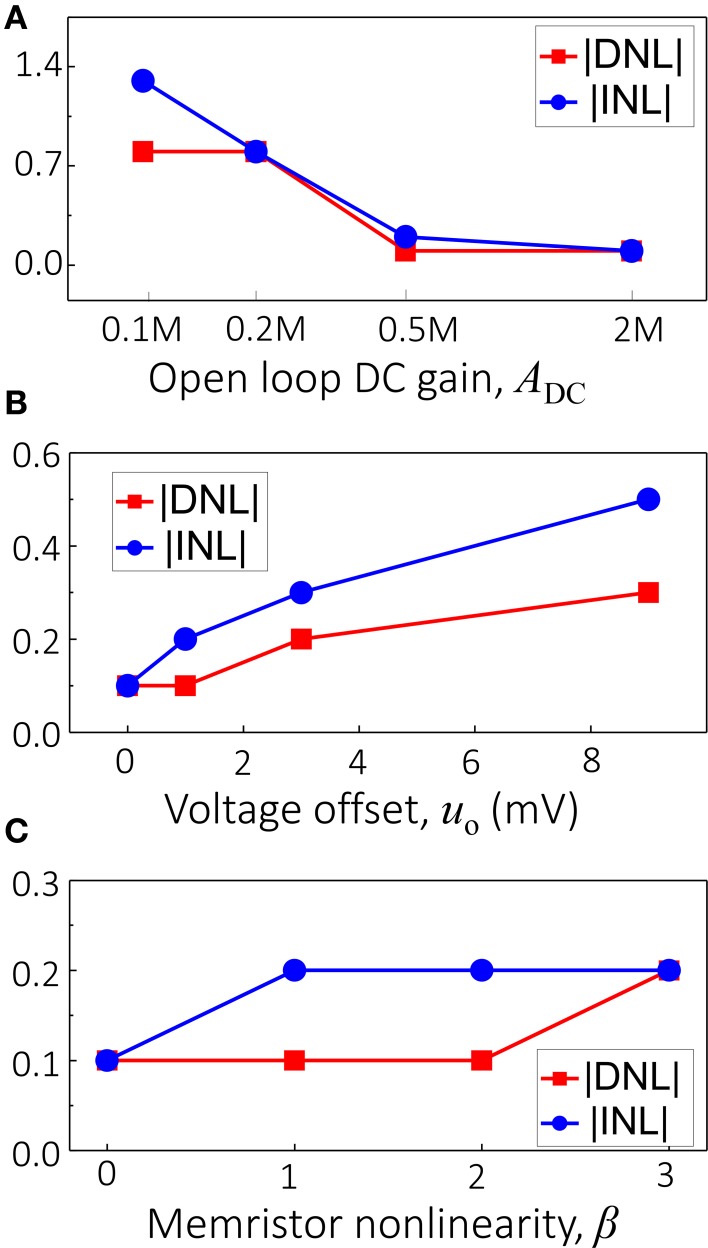
**Theoretical analysis of the performance sensitivity of a 4-bit Hopfield network ADC with respect to (A) open-loop DC gain, (B) voltage offsets in the operational amplifiers, and (C) the nonlinearity of memristive devices**.

Using Equation (8) for the memristors and SPICE models for the IC components, in the next series of simulations we studied how particular non-ideal behavior affects differential (DNL) and integral (INL) nonlinearities in ADC circuits (van de Plassche, 2003). Figure 3A shows INL and DNL as a function of the open loop DC gain, which was varied simultaneously for all three op-amps, assuming ideal memristors with β = 0 and no voltage offset. Note that in this simulation, the gain-bandwidth product (*GBP*) was increased proportionally to the open loop DC gain, and was equal to 3 MHz at $A_{\text{DC}}=2\times 10^{5}$. Because the circuit operated at about 1.5 KHz, the effective gain *A* ≈ *A*_DC_/100 for all simulations (and also for the experimental work discussed below). Figure 3B shows the impact of the voltage offset on DNL and INL (simulated as an offset on the ground nodes), which was varied simultaneously for all three op-amps. Finally, Figure 3C shows the effect of *I-V* nonlinearity, which was varied by changing constant β in Equation (8), assuming all other parameters of the network to be close to ideal, i.e., that the voltage offset *u*_o_ = 0 and the open loop DC gain $A_{\text{DC}}=10^{6}$. Note that for β > 0, the memristor weights were chosen in such a way that the conductance of the device at −0.2V matched the corresponding values prescribed by Equation (10).

**Figure 4:**
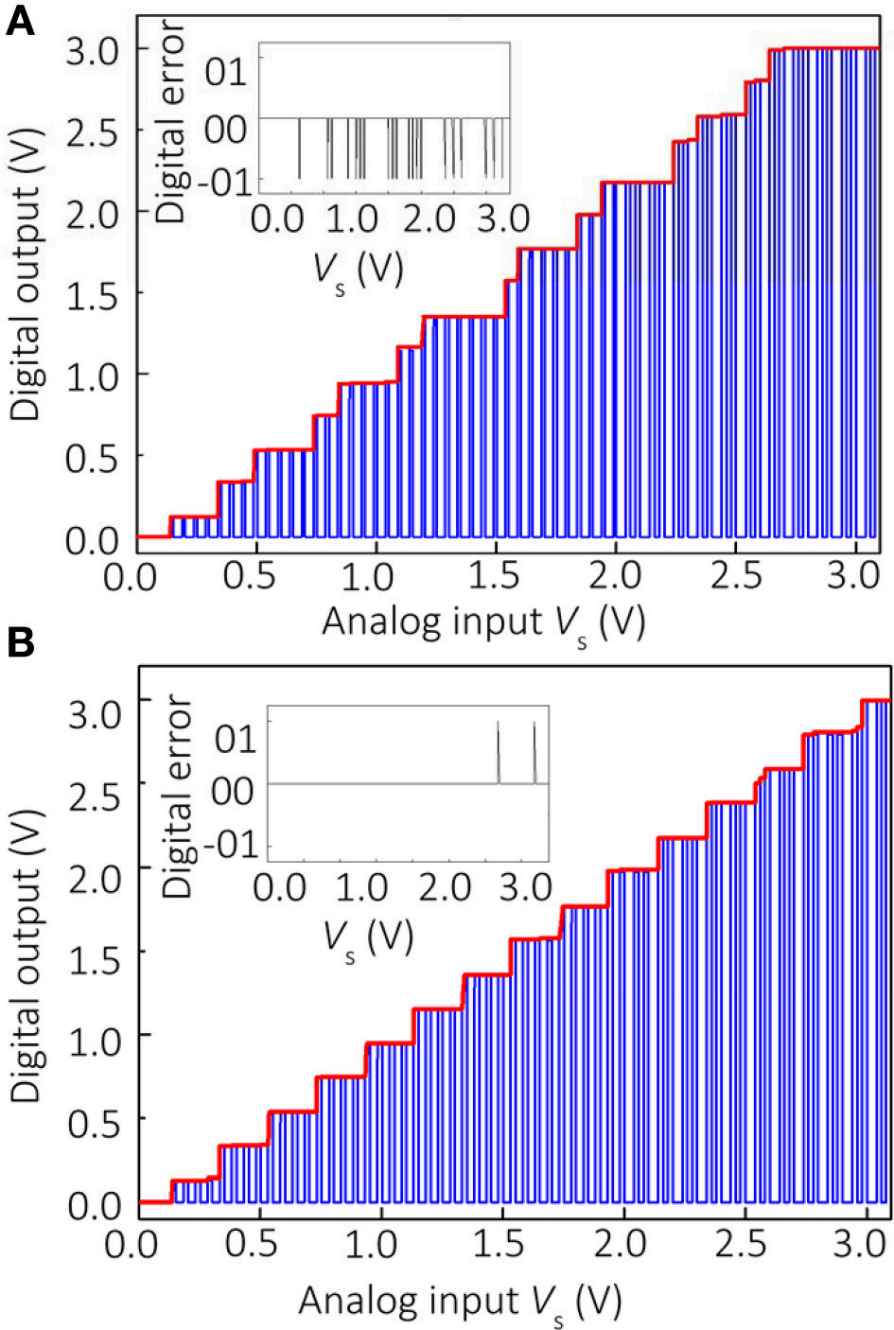
**Simulation results for (A) the original and (B) the optimized 4-bit Hopfield network ADC with β = 1, $A_{\text{DC}}=2\times 10^{5}$, and *u*_o_ = 3 mV voltage offset, which are representative parameters for the experimental setup**. For the optimized network, *T*_R_” = 0.97 *T*_R1_, *T*_R2_” = 0.86 *T*_R2_, *T*_R3_” = 0.95 *T*_R3_, *T*_R4_” = 0.97 *T*_R4_.

**Figure 5:**
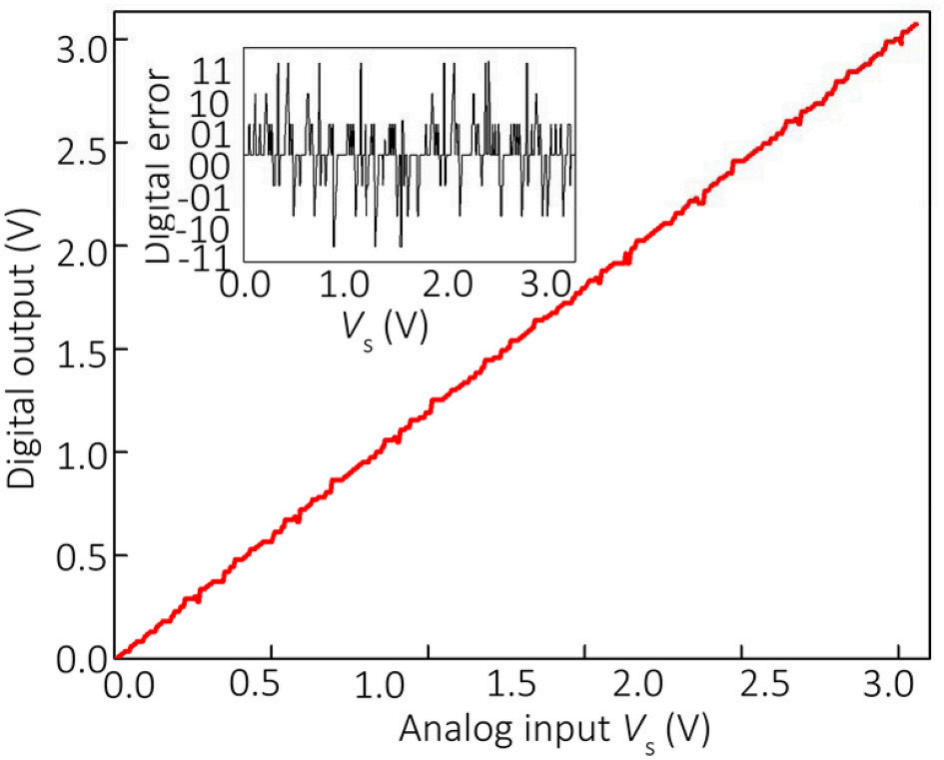
**Simulation results for the optimized 8-bit Hopfield network ADC with *T*_R1_” = 0.8 *T*_R1_, *T*_R2_” = 0.81 *T*_R2_, *T*_R3_” = 0.89 *T*_R3_, *T*_R4_” = 0.83 *T*_*R*4_, *T*_R5_” = 0.55 *T*_R5_, *T*_R6_” = 0.74 *T*_R6_, *T*_R7_” = 0.71 *T*_R7_, *T*_R8_” = 0.75 *T*_R8_**. All other parameters are equal to those used for Figure 4.

The results shown in Figure 3 confirm the significant individual contribution of the considered sources of non-ideal behavior on the ADC's performance. Figure 4A shows the simulation results considering all these factors together for the specific values *u*_o_ = 3 mV, β = 1, $A_{\text{DC}}=2\times 10^{5}$, and *GBP* = 3 MHz, which are representative of the experimental setup. The gain and voltage offset values were taken from the specifications of the discrete IC op-amps used in the experiment. Clearly, the ADC output is distorted and contains numerous errors, with the largest contribution to INL being due to finite gain (Figure 3). Figures 4B, 5 show the simulation results with new values for the reference weights calculated according to Equation (11) for the 4-bit and 8-bit ADCs, respectively. The results shown in these figures confirm that non-ideal behavior in op-amps, such as limited gain and voltage offsets, can be efficiently compensated by fine-tuning memristors.

### Experimental results

**Figure 6:**
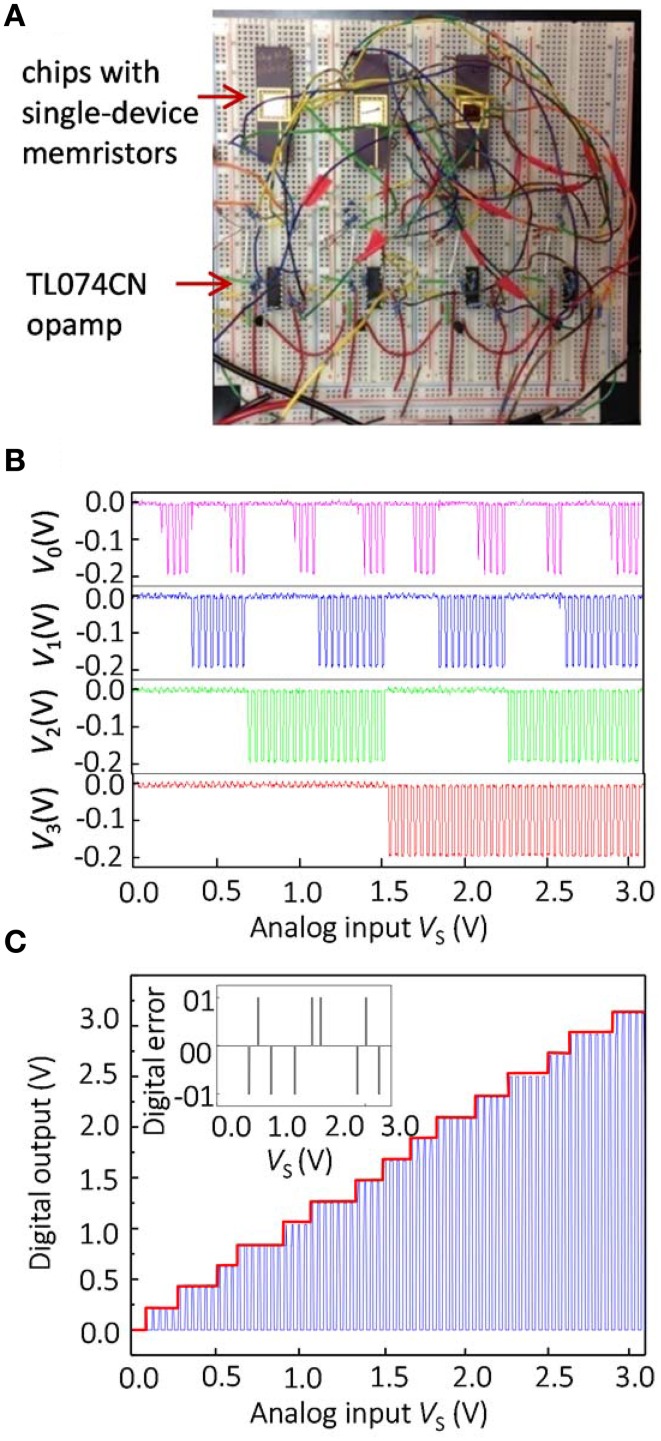
**Experimental results for the optimized 4-bit Hopfield ADC: (A) experimental setup, (B) measured outputs for every output channel, and (C) measured transfer characteristics**.

The simulation results were also validated experimentally by implementing a 4-bit Hopfield network ADC in a breadboard setup consisting of Pt/TiO_2−x_/Pt memristive devices and discrete IC CMOS components (Figure 6A). The memristor chips were assembled in standard 40-pin DIP packages by wire-bonding 20 standalone memristive devices. Because input voltage range is 0 ≤ *V*_S_ ≤ $V_{\text{S}}^{\text{max}}=3.0$ V, the weights *T*_S_ were realized with regular resistors^2^. The discrete memristors and other IC components were then connected as shown in Figure 1 with external wires.

The memristors implementing feedback and reference weights were first tuned ex-situ using a previously developed algorithm (Alibart et al., 2012) to the values defined by Equation (10). The ex-situ tuning for each memristor was performed individually before the devices were connected in a circuit. This was done to simplify the experiment and it is worth mentioning that in general, it should be possible to tune memristors after they are connected in the crossbar circuit, as it was experimentally demonstrated by our group for standalone devices connected in crossbar circuits (Alibart et al., 2013; Gao et al., 2013c) and integrated passive crossbar circuits (Prezioso et al., 2015a,b).

As was discussed in Sections Materials and Methods for Hopfield Network Implementation with Hybrid Circuits and Results, limited gain and voltage offsets of operational amplifiers can be compensated by adjusting reference weights according to Equations (11, A12). To demonstrate in-field configurability of memristors, the reference weights were fine-tuned in-situ. In particular, reference weights were adjusted to ensure correct outputs at four particular input voltages, when *V*_S_ is equal to 1/16, 1/8, 1/4, and 1/2 of its maximum value. The tuning is performed first for *V*_S_ = 1∕16 $V_{\text{S}}^{\text{max}}$, for which the correct operation of ADC assumes that the least significant output bit *V*_0_ flips from 0 to 1 (corresponding to voltage 0.2 V in our case), which is ensured by fine-tuning reference weight *T*_R0_. Similarly, the output bit *V*_1_ should flip from 0 to 1 when *V*_S_ = 1∕8 $V_{\text{S}}^{\text{max}}$, which is ensured by fine-tuning reference weight *T*_R1_ and so on. Because we started fine-tuning from the least significant output, it is sufficient to fine-tune only one corresponding reference weight at a time for a particular input voltage, which greatly simplified in-situ tuning procedure. Also, the direction of adjustment was always straightforward to determine due to monotonic dependence of the input voltage at which a particular output bit flips from 0 to 1, on the corresponding reference weight (Equation 11).

## Discussion

The network parameters for the experimental work are summarized in Table 1. Although there were a few A/D conversion errors in the experimental work (Figure 6), the results are comparable with the simulations of the optimized network, and much better than those obtained for the unoptimized network. The experimental results for the unoptimized network were significantly worse in comparison with the simulation, and are not shown in this paper.

**Table 1:** **Parameters for the experimentally demonstrated Hopfield network ADC**.

**Feed-back**	**Conductance (S@0.2V)**	**Reference**	**Conductance (S@0.2V)**
*T*2,1	2e-5	*T*_1R_	4.75e-6
*T*3,1	4e-5	*T*_2R_	2.19e-5
*T*4,1	7.9e-5	*T*_3R_	9.33e-5
*T*1,2	2e-5	*T*_4R_	41.85e-5
*T*3,2	7.9e-5	Input	Conductance (S)
*T*4,2	15e-5	*T*_1S_	8.33e-6
*T*1,3	4e-5	*T*_2S_	1.67e-5
*T*2,3	7.9e-5	*T*_3S_	3.33e-5
*T*4,3	30.9e-5	*T*_4S_	6.67e-5
*T*1,4	7.9e-5	Neuron	Conductance (S)
*T*2,4	15e-5	*T*_N1_	1e-3
*T*3,4	30.9e-5	*T*_N2_	1e-5
		*T*_N3_	5e-4

It is worth mentioning that for the considered memristors drift of conductive state over time was negligible due to highly nonlinear switching kinetics specific to these devices (Alibart et al., 2012, 2013; Prezioso et al., 2015a). In principle, for other types of memristors with inferior retention properties it should be possible to occasionally fine-tune memristor state to cope with conductance drift. A related issue might be measurement noise upon reading the state of the memristor, e.g., due to the fluctuations in the device conductance over time, which is sometimes observed as random telegraph noise (Gao et al., 2012, 2013b; Prezioso et al., 2015b). Such noise can be tolerated by performing quasi DC read measurements, however, the downside would be potentially much slower tuning process.

To conclude, in this work we investigated hybrid CMOS/metal-oxide-memristor circuit implementation of a Hopfield recurrent neural network performing analog-to-digital conversion tasks. We showed that naïve implementation of such networks, with weights prescribed by the original theory, produces many conversion errors, mainly due to the non-ideal behavior of the CMOS components in the integrated circuit. We then proposed a method of adjusting weights in the Hopfield network to overcome the non-ideal behavior of the network components and successfully validated this technique experimentally on a 4-bit ADC circuit. The ability to fine-tune the conductances of memristors in a circuit was essential for implementing the proposed technique. In our opinion, the work carried out proved to be an important milestone and its results will be valuable for implementing more practical large-scale recurrent neural networks with CMOS/memristor circuits. Experimental research into CMOS/memristor neural networks is still very scarce and, to the best of our knowledge, the demonstrated Hopfield network is the most complex network of its type reported to date. From a broader perspective, this paper demonstrates one of the main advantages of utilizing memristors in analog circuits, namely the feasibility of fine-tuning memristors after fabrication to overcome variations in analog circuits.

## Author contributions

XG and FMB performed simulation work. FMB, XG, and LG performed the experimental demo. LG, BH, and FA fabricated devices. DS supervised the project. All discussed the results.

### Conflict of interest statement

The authors declare that the research was conducted in the absence of any commercial or financial relationships that could be construed as a potential conflict of interest.

## Footnotes

^1^The sign of the first term on the left in Equation (2a), and of all right hand terms in Equation (5), is different from that of the original paper (Hopfield, 1984). In this work we assume that all weights are strictly positive, making it necessary explicitly to flip the neuron feedback signal sign.

^2^In principal, input voltage range could be decreased by increasing input weights correspondingly. However, such rescaling would require larger a dynamic range of conductances to implement (Equation 6), and this was not possible with the considered memristive devices.

## Appendix

Assuming negligible op-amp input currents and output impedances, the Hopfield network is described by the following equations, which also account for limited gain and voltage offsets:

(A1) $$\begin{array}{rcl} V_{xj} & = & A_{1j}\left(u_{o1j}-V_{inj}\right), \end{array}$$

(A2) $$\begin{array}{rcl} V_{zj} & = & g\left(u_{o2j}+U_{j}\right), \end{array}$$

(A3) $$\begin{array}{rcl} -V_{j} & = & A_{3j}\left(u_{o3j}-V_{wj}\right), \end{array}$$

(A4) $$\begin{array}{l} T_{\text{N}1}\left(V_{inj}-V_{xj}\right)=T_{\text{R}j}\left(-V_{\text{R}}-V_{inj}\right)+T_{\text{S}j}\left(V_{\text{S}}-V_{inj}\right)\\ \;+\sum_{i}T_{ij}(-V_{i}-V_{inj}) \end{array}$$

(A5) $$\begin{array}{rcl} -C{\dot{U}}_{j} & = & T_{\text{N}1}\left(V_{xj}+U_{j}\right), \end{array}$$

(A6) $$\begin{array}{rcl} T_{N2}\left(V_{\text{z}j}-V_{\text{w}j}\right) & = & T_{\text{N}3}\left(V_{\text{w}j}+V_{j}\right). \end{array}$$

Solving these equations results in the following dynamic equation

(A7a) $$a_{j}C{\dot{U}}_{j}^{\prime }\;=-\sum_{i}T_{ij}V_{i}^{\prime }-a_{j}T_{\text{N}1}U_{j}^{\prime }+I_{j}+I_{oj}$$

(A7b) $$b_{j}V_{j}^{\prime }=g\left(U_{j}^{\prime }\right),$$

where *g*() is a transfer function of the saturating amplifier implemented with the second op-amp, and

(A8) $$U_{j}^{\prime }=u_{o2j}+U,$$

(A9) $$V_{i}^{\prime }{}_{i}=u_{o3j}(1+T_{\text{N}3j}/T_{\text{N}2j})/b_{j}+\;V_{i},$$

(A10) $$\begin{array}{rcl} a_{j} & = & 1+\left(1+T_{j}∕T_{\text{N}1j}\right)∕A_{1j}, \end{array}$$

(A11) $$\begin{array}{rcl} b_{j} & = & {T{}_{\text{N}3}}_{j}∕T_{\text{N}2j}+\left(1+T_{\text{N}3j}∕{T{}_{\text{N}2}}_{j}\right)∕A_{3j}, \end{array}$$

(A12) $$\begin{array}{l} I_{oj}=-\left(T_{\text{N}1j}+T_{j}\right)u_{o1j}+a_{j}T_{\text{N}1j}u_{o2j}\\ \;+\;\frac{1+\frac{T_{\text{N}3j}}{T_{\text{N}2j}}}{b_{j}}\sum_{i}T_{ij}u_{o3j} \end{array}$$
